# Genetic variations of NTCP are associated with susceptibility to HBV infection and related hepatocellular carcinoma

**DOI:** 10.18632/oncotarget.22211

**Published:** 2017-10-31

**Authors:** Peng Wang, Ruidong Mo, Rongtao Lai, Yumin Xu, Jie Lu, Gangde Zhao, Yuhan Liu, Zhujun Cao, Xiaolin Wang, Ziqiang Li, Lanyi Lin, Huijuan Zhou, Wei Cai, Hui Wang, Shisan Bao, Xiaogang Xiang, Qing Xie

**Affiliations:** ^1^ Department of Infectious Diseases, Ruijin Hospital, Shanghai Jiaotong University School of Medicine, Shanghai, China; ^2^ Translational Lab of Liver Diseases, Department of Infectious Diseases, Ruijin Hospital, Shanghai Jiaotong University School of Medicine, Shanghai, China; ^3^ Discipline of Pathology, School of Medical Sciences and Bosch Institute, The University of Sydney, New South Wales 2006, Australia

**Keywords:** Na^+^ taurocholate cotransporting polypeptide, association study, HBV, HCC, meta-analysis

## Abstract

Sodium taurocholate cotransporting polypeptide (NTCP), encoded by gene SLC10A1, is a receptor for hepatitis B virus (HBV). The aim of the current study was to investigate the role of NTCP polymorphisms in HBV susceptibility, cirrhosis and hepatocarcinogenesis. A total 1221 cases [including 866 chronic hepatitis B (CHB), 238 liver cirrhosis (LC), 117 hepatocellular carcinoma (HCC) patients] and 1232 healthy controls (HCs) were recruited, and 6 single nucleotide polymorphisms (SNPs) were genotyped. Meta-analysis was executed among 14591 CHBs and 12396 HCs to determine the association between NTCP polymorphisms and HBV infection, cirrhosis or hepatocarcinogenesis. The frequency of rs2296651-GA was inversely correlated with CHB, LC or HCC patients [adjusted OR(95%CI)=0.16(0.11-0.23), *p*<0.001; 0.34(0.21-0.55), *p*=0.001; or 0.46(0.25-0.83), *p*=0.008], respectively, compared with HCs. Meta-analysis also showed that NTCP rs2296651-GA was inversely associated with HBV infection [OR(95%CI)=0.532(0.287-0.986), *p*=0.028, codominant] or HBV-related HCC [OR(95%CI)=0.701(0.564-0.872), *p*=0.001, recessive]. Furthermore, the frequency of rs943277-GA was positively correlated with HBV infection [adjusted OR(95%CI)=2.42(1.05-5.54), *p*=0.032, codominant]. Our data suggest that NTCP mutants contribute to the susceptibility of HBV infection or HBV-related HCC.

## INTRODUCTION

Chronic hepatitis B (CHB) is still an important challenge to the clinician due to high morbidity (∼350 million) and mortality (∼800k a year) around the world [1]. The prevalence of hepatitis B virus (HBV) infection remains high in China (7.18%) [2], with ∼120 million chronic HBV carriers [3]. The risk for hepatocellular carcinoma (HCC) in chronic HBV carriers is approximate 40 times higher than that in non-carriers. Furthermore, a large proportion of CHB patients progress into irreversible liver cirrhosis or HCC [4, 5].

Recently, sodium taurocholate cotransporting polypeptide (NTCP) found on hepatocytes has been considered as a key receptor for HBV infection [6]. NTCP, encoded by the gene SLC10A1, expressed at the basolateral (sinusoidal) membrane of hepatocyte [7], is a determinant for HBV cellular entrance by binding the pre-S1 domain of HBV large envelope proteins [6, 8]. NTCP is down-regulated by cyclin D1 in HBV-related HCC, especially in HCC patients with a poor prognosis [9].

The relationship between NTCP polymorphisms and HBV infection or HCC is controversial [10–14]. However, it remains to be explored whether NTCP polymorphisms influence the susceptibility of HBV infection and the occurrence of liver cirrhosis (LC) or HCC. Therefore, the present study aimed to evaluate the role and clinical relevance of NTCP polymorphisms in HBV susceptibility and HBV-related HCC, especially in the Chinese Han population, using experimental date linked to meta-analysis.

## RESULTS

### Demographic and clinical characteristics

The general demographic characteristics of the study population are shown in Table 1. In total, 2453 participants were identified in the study, including 1221 CHB patients (866 CHB without LC and HCC, 238 CHB with LC, 117 CHB with HCC) and 1232 HCs (Table 1). The mean age of CHB patients was similar to HCs (46.24±13.28 vs 46.51±13.82 years, *p*=0.419). The CHB group had a higher, but not significant, male/female ratio than HC (70.52% vs 58.36%, *p*= 0.077). The levels of serum alanine aminotransferase (ALT), aspartate aminotransferase (AST) and alpha-fetoprotein (AFP) in the CHB group were significantly higher than that in HCs (*p<*0.0001). In addition, the quantity of HBV DNA in the CHB group (patients without LC or HCC) was higher than that in LC group or with HCC group (*p*= 0.006) (Table 1).

**Table 1:** Demographic and clinical features of the patients and healthy controls in the study

Characteristic	HC (n=1232)	Total (n=1221)	I	II	III	*p*-value^‡^
			CHB without LC and HCC (n=866)	CHB with LC (n=238)	CHB With HCC (n=117)	
**Mean Age**^†^	46.51±13.81	46.24±13.28	44.16±13.72	49.68±10.40	54.60±10.22	0.4188
Discover group	42.18±12.45	43.72±14.85	43.17±15.19	46.79±10.57	52.80±14.87	0.407
Replication group	47.74±13.81	46.89±12.78	44.50±13.18	50.00±10.36	54.68±10.06	0.16
**Gender** (n, %) (Male/Female)	719(58.36)/ 513(41.64)	861(70.52)/ 360(29.48)	581(67.09)/ 285(32.93)	182(76.47)/ 56(23.53)	98(83.76)/ 19(16.24)	0.077
Discover group	133(50.95)/ 128(49.05)	134(53.39)/ 117(46.61)	110(49.54)/ 112(50.46)	20(83.33)/ 4(16.67)	4(80.00)/ 1(20.00)	0.185
Replication group	586(60.35)/ 385(39.65)	727(74.94)/ 243(25.06)	471(73.13)/ 173(26.87)	162(74.65)/ 52(25.35)	94(83.92)/ 18(16.08)	0.075
**ALT**^†^	20.52±9.166	270.8±431.2	341.0±488.1	110.6±158.0	87.21±163.8	<0.0001
Discover group	22.15±9.217	300.9±466.5	335.2±499.7	70.75±43.40	97.40±70.07	<0.0001
Replication group	20.08±9.108	263.1±421.5	342.9±484.6	115.1±165.5	86.76±166.9	<0.0001
**AST**^†^	19.35±4.969	175.7±233.9	200.9±263.4	116.5±125.4	110.2±119.3	<0.0001
Discover group	21.14±4.706	184.8±260.9	197.9±279.3	77.13±36.84	117.2±91.42	<0.0001
Replication group	18.87±4.929	173.4±226.5	201.9±258.2	121.0±131.0	109.9±120.7	<0.0001
**AFP**^†^	1.945±1.715	409.1±2336	159.5±779.2	182.4±367.9	2262±6226	<0.0001
Discover group	1.641±1.237	217.8±1086	149.2±823.8	93.99±186.6	3656±4543	<0.0001
Replication group	2.008±1.749	459.8±2554	162.7±765.7	229.4±410.7	2205±6296	<0.0001
**HBV-DNA** (copies/ml)		(1.22±5.55)×10^7	(1.58±6.5)×10^7	4.07×10^6±1.56×10^7	2.45×10^6±1.18×10^7	0.002
>100000 (n, %)		519(42.5)	411(47.5)	81(33.8)	31(26.4)	
Discover group		(1.97±7.03)×10^7	(2.18±7.41)×10^7	8.6×10^5±2.78×10^6	9.71×10^6±1.11×10^7	0.394
>100000 (n, %)		137(54.6)	122(55.0)	11(47.8)	4(80)	
Replication group		(1.03±5.1)×10^7	(1.37±6.15)×10^7	4.4×10^6±1.63×10^7	2.12×10^6±1.11×10^7	0.013
>100000 (n, %)		366(37.7)	279(43.3)	68(31.9)	25(22.1)	
**eAg +** (n,%)	/	424(34.72)	320(36.95)	72(30.25)	33(28.20)	/
Discover group	/	116(46.21)	104(46.84)	9(37.5)	3(60.00)	/
Replication group	/	309(31.86)	216(33.54)	63(29.43)	30(26.78)	/

CHB: chronic hepatitis B; LC: HBV-related liver cirrhosis; HCC: HBV-related hepatocellular carcinoma; HC: health control; ^†^Date presented as (Mean ± SD); ^‡^*P*-value: *x^2^* test for all of all of CHB patients compared to HC individuals; Total group is the sum of group I, group II and group III.

### Quality assessment

In total, 6928 variants of 6 loci were successfully genotyped in the 2453 samples. The rates of successful genotyping (call rate) ranged from 96.5% to 100% (Supplementary Table 2). Hardy-Weinberg disequilibrium was assessed, using the HaploView 4.2 test. The genotype distribution of these 6 SNPs was in accord with the Hardy-Weinberg equilibrium in the HCs (Supplementary Table 2). Based on applying these quality control measures, these results were suitable for genetic analysis.

### Inverse association between NTCP rs2296651 mutant and HBV susceptibility

The minor allele frequency (MAF) was compared between CHB patients and HCs. The frequency of the minor A allele at rs2296651 in CHB patients was significantly lower than that of HCs in discovery group [OR(95%CI)=0.19(0.09-0.38), *p*<0.001] (Table 2). A different frequency distribution of the rs2296651 mutant between CHB and HCs was also found in the replication group [OR(95%CI)=0.20(0.14-0.93), *p*<0.001]. In total, the frequency of A allele at rs2296651 in CHB patients was inversely correlated with HBV infection [OR(95%CI)=0.19(0.14-0.26), *p*<0.001] (Table 2).

**Table 2:** Association between rs2296651, rs943277 and HBV infection in Discovery and Replication group

Rs ID	Model	Genotype	Discovery group			*P*_I_	Replication group			*P*_II_	Total			*P*_T_
			CHB Patients	Controls	AOR_I_(95% CI)		CHB Patients	Controls	AOR_II_(95% CI)		CHB Patients	Controls	AOR_T_(95% CI)	
rs2296651	Alleles	G	492 (98.0)	455 (90.3)	1		1871 (97.7)	1717(89.1)	1		2363(97.7)	2172(89.3)	1	
		A	10 (2.0)	49 (9.7)	**0.19 (0.09-0.38)**	**<0.001**	45 (2.3)	211(10.9)	**0.20 (0.14-0.93)**	**<0.001**	55(2.3)	260(10.7)	**0.19 (0.14-0.26)**	**<0.001**
	Codominant	GG	241(96.0)	203(80.6)	1		915(95.5)	753(78.1)	1		1156(95.6)	956(78.6)	1	
		GA	10(4.0)	49(19.4)	**0.17 (0.09-0.35)**	**<0.001**	41(4.3)	211(21.9)	**0.16 (0.12-0.22)**	**<0.001**	51(4.2)	260(21.4)	**0.16 (0.11-0.23)**	**<0.001**
		AA	0(0)	0(0)	NA	NA	2(0.2)	0(0)	NA	NA	2(0.2)	0(0)	NA	NA
	Dominant	GG+GA	251(100)	252(100)	NA	NA	956(99.8)	964(100)	NA	NA	1207(99.8)	1216(100)	NA	NA
		AA	0(0)	0(0)	NA	NA	2(0.2)	0(0)	NA	NA	2(0.2)	0(0)	NA	NA
	Recessive	AA+GA	10(4.0)	49(19.4)	1		43(4.5)	211(21.9)	1		53(4.4)	260(21.4)	1	
		GG	241(96.0)	203(80.6)	**0.17 (0.08-0.34)**	**<0.001**	915(95.5)	753(78.1)	**0.19 (0.14-0.28)**	**<0.001**	1156(95.6)	956(78.6)	**0.18 (0.13-0.25)**	**<0.001**
	Additive				**0.17 (0.08-0.37)**	**<0.001**			**0.21 (0.15-0.29)**	**<0.001**			**0.19 (0.16-0.26)**	**<0.001**
rs943277	Alleles	G	485 (97.0)	513 (99.0)	1		1932 (99.7)	1933 (99.8)	1		2417(99.1)	2446(99.7)	1	
		A	15 (3.0)	5 (1.0)	**3.17 (1.14-8.80)**	**0.019**	6(0.30)	3 (0.20)	2.0 (0.50-8.02)	0.318	21(0.9)	8(0.3)	**2.66 (1.17-6.01)**	**0.014**
	Codominant	GG	236 (94.4)	254(98.1)	1		963(99.4)	965(99.7)	1		1199(98.4)	1219(99.3)	1	
		GA	13 (5.2)	5(1.9)	**2.80 (0.98-7.97)**	**0.045**	6(0.60)	3(0.30)	2.0 (0.50-8.04)	0.317	19(1.6)	8(0.7)	**2.42 (1.05-5.54)**	**0.032**
		AA	1 (0.4)	0(0)	0.31 (0.01-7.65)	0.3	0(0)	0(0)	NA	NA	1(0)	0(0)	NA	NA
	Dominant	GG+GA	249 (99.6)	259(100.0)	1		969(100.0)	968(100.0)	NA	NA	1218(99.9)	1227(100)	NA	NA
		AA	1 (0.4)	0(0)	NA	NA	0(0)	0(0)	NA	NA	1(0.1)	0(0)	NA	NA
	Recessive	AA+GA	14(5.6)	5(1.9)	1		6(0.60)	3(0.30)	1		20(1.6)	8(0.7)	1	
		GG	236(94.4)	254(98.1)	**3.22 (1.13-9.19)**	**0.028**	963(99.4)	965(99.7)	1.92 (0.47-7.89)	0.365	1199(98.4)	1219(99.3)	**2.32 (1.01-5.34)**	**0.048**
	Additive				**3.17 (1.13-8.84)**	**0.027**			1.92 (0.47-7.89)	0.365			**2.34 (1.05-5.22)**	**0.039**

Data were presented as number (percentage) for every group. The differences of genotype frequencies among groups were analyzed using logistic regression models (Codominant, Recessive, Dominant, additive). Age and sex were included as covariates. AORs (adjusted odds ratio) were calculated and reported within the 95% CI (confidence interval). AOR_I_, AOR_II_ and AOR_T_ were calculated for discovery group, replication group and total of patients, respectively. Significant p-values (*p* < 0.05) are highlighted in bold.

Subsequently, genetic models (codominant, dominant, recessive and additive) were applied to calculate genotype frequencies. A multivariate logistic regression analysis was performed to identify the variation on rs2296651 independently associated with HBV infection. Age and gender covariates were included in the logistic regressions, which has been previously reported to be significantly associated with HBV infection [15]. In the codominant model, the frequency of GA genotypes in CHB patients accounted for a relatively small proportion of effect (Adjusted OR_I_=0.17, *p<*0.001 and Adjusted OR_II_=0.16, *p<*0.001, adjusted by logistic regression), compared to that of HCs (Table 2). Similarly, the frequency of GA+AA genotypes in the recessive model in CHB patients was significantly lower than that in HCs (4% vs 19.4%, AOR_I_=0.17, *p*<0.001) in the discovery group. The trend was verified again in the replication group (4.9% *vs* 21.9%, AOR_II_=0.19, *p*<0.001) (Table 2). Moreover, in the additive model, the frequencies of GA and AA genotypes at rs2296651 in CHB patients were significantly lower than HCs in both the AOR_I_ (discovery group) and the AOR_II_ (replication group), being 0.19 (*p*<0.001) or 0.20 (*p*<0.001), respectively (Table 2).

Finally, the association between the rs2296651 mutant and HBV infection based on the merged data of the two groups was illustrated. Genetics analysis demonstrated that the rs2296651 mutant was inversely associated with HBV susceptibility, regardless of allele frequency (OR=0.19, *p*<0.001), codominant model (AOR=0.16, *p*<0.001), dominant model (AOR=0.18, *p*<0.001) or additive model (AOR=0.19, *p*<0.001) (Table 2). The rs2296651 mutant was inversely correlated with HBV susceptibility.

### Association between the rs943277 mutant and HBV susceptibility

In the discovery group, the A allele for rs943277 was significantly higher in CHB patients than that in HCs (3.0% vs 1.5%, OR=3.17, 95%CI=1.14-8.8, *p*=0.019) (Table 2). In logistic regression analysis, the genotype frequencies of GA and AA were all significantly higher in CHB patients, using a codominant model (AOR_I_=2.80, *p*=0.045), dominant model (AOR_I_=3.22, *p*=0.028) and additive model (AOR_I_=3.17, *p*=0.027) (Table 2). In the replication group study, there was no significant statistical difference for the rs943277 mutant among CHB patients and HCs. The values of AOR_II_ were 2.0 (*p*=0.317), 1.92 (*p*=0.365) and 2.0 (*p*=0.318), in codominant, dominant and additive genetics model, respectively (Table 2). When the data for the two groups were merged, the difference for the rs943277 mutant between CHB and HCs was statistically significant (AOR=2.66, 95%CI=1.17-6.01, *p*= 0.014) (Table 2).

### Other SNPs and HBV susceptibility

As for rs17556915, rs9323529, rs943276 and rs4646296, there were no significant differences between CHBs and HCs in the discovery group, regardless of comparing allele frequencies or genotypes (Table 3). Considering the lower minor allele frequency in CHBs and HCs (1.0% vs 0.4%, 0.4% vs 0.8%, 0.2% vs 0.8%, and 9.2% vs 8.1%), and non-statistically significant differences between the two groups (all *p*>0.05), it was not justified to further verify these SNP sites in the replication group (Table 3).

**Table 3:** Association between 4 loci of NTCP mutant and HBV infection in discovery group

Rs ID	Model	Genotype	Patients	Control	AOR(95% CI)	*p*
rs17556915	Alleles	G	495 (99.0)	520 (99.6)	1	
		A	5 (1.0)	2 (0.4)	2.63 (0.507-13.6)	0.232
	Codominant	GG	245(98.0)	259(99.2)	1	
		GA	5(2.0)	2(0.8)	2.643 (0.5078-13.75)	0.23
		AA	0(0)	0(0)	NA	NA
	Dominant	GG+GA	250(100.0)	261(100.0)	1	
		AA	0(0)	0(0)	NA	NA
	Recessive	AA+GA	5(2.0)	2(0.8)	1	
		GG	245(98.0)	259(99.2)	2.83(0.537-14.9)	0.22
	Additive				2.83(0.537-14.9)	0.22
rs4646296	Alleles	G	452 (90.8)	478 (91.9)	1	
		C	46 (9.2)	42 (8.1)	1.16 (0.748-1.79)	0.51
	Codominant	GG	205 (82.0)	220(84.6)	1	
		GC	44 (17.6)	38(14.6)	1.243 (0.774-1.99)	0.368
		CC	1 (0.4)	2(0.8)	1.86 (0.168-20.72)	0.607
	Dominant	GG+GC	249 (99.6)	258(99.2)	1	
		CC	1(0.4)	2 (0.8)	0.441(0.039-4.98)	0.508
	Recessive	CC+GC	45 (18.0)	40 (15.4)	1	
		GG	205 (82.0)	220 (84.6)	1.16(0.720-1.86)	0.547
	Additive				1.11(0.705-1.73)	0.661
rs9323529	Alleles	T	500 (99.6)	516 (99.2)	1	
		G	2 (0.4)	4 (0.8)	0.516 (0.094-2.83)	0.438
	Codominant	TT	249 (99.2)	256 (98.5)	1	
		TG	2 (0.8)	4(1.5)	0.514 (0.093-2.83)	0.44
		GG	0 (0)	0(0)	NA	NA
	Dominant	TT+TG	251 (100.0)	260(100.0)	1	
		GG	0 (0)	0(0)	NA	NA
	Recessive	GG+TG	2 (0.8)	4(1.5)	1	
		TT	249 (99.2)	256(98.5)	0.537(0.097-2.98)	0.477
	Additive				0.537(0.097-2.98)	0.477
rs943276	Alleles	G	499 (99.8)	514 (99.2)	1	
		A	1 (0.2)	4 (0.8)	0.258 (0.029-2.31)	0.191
	Codominant	GG	249(99.6)	255(98.5)	1	
		GA	1(0.4)	4(1.5)	0.256 (0.028-2.31)	0.191
		AA	0(0)	0(0)	NA	NA
	Dominant	GG+GA	250(100.0)	259(100.0)	1	
		AA	0(0)	0(0)	NA	NA
	Recessive	AA+GA	1(0.4)	4(1.5)	1	
		GG	249(99.6)	255(98.5)	0.268(0.029-2.44)	0.242
	Additive				0.268(0.029-2.44)	0.242

AOR, adjusted odds ratio; CI, confidence interval. Data were presented as number (percentage) for every group. The differences in genotype frequencies between any two groups were analyzed using logistic regression models (Codominant, Recessive, Dominant, additive). Age and sex were included as covariates. AORs were calculated and reported within the 95% CI. Significant p-values (*p* < 0.05) are highlighted in bold.

### Meta-analysis of the association between NTCP polymorphisms and HBV susceptibility

Based on our current data and systematic review of other studies up to date, meta-analysis was adopted to illuminate the association between these host genetic mutants of NTCP and HBV susceptibility. In total, 9 studies were extracted for meta-analysis, including 7 studies [10–14, 16, 17] and our data focused on rs2296651, 4 studies [13, 14, 16, 18] on rs4646287, 4 studies [14, 16, 18, 19] on rs7154439 and 2 studies [14, 18] linked with our data on rs4646296. Quality assessment showed the high quality of the included studies. The host genetics characteristics as well as the quality scores of the included studies are illustrated in Supplementary Table 4 and Supplementary Table 3.

**Figure 1:**
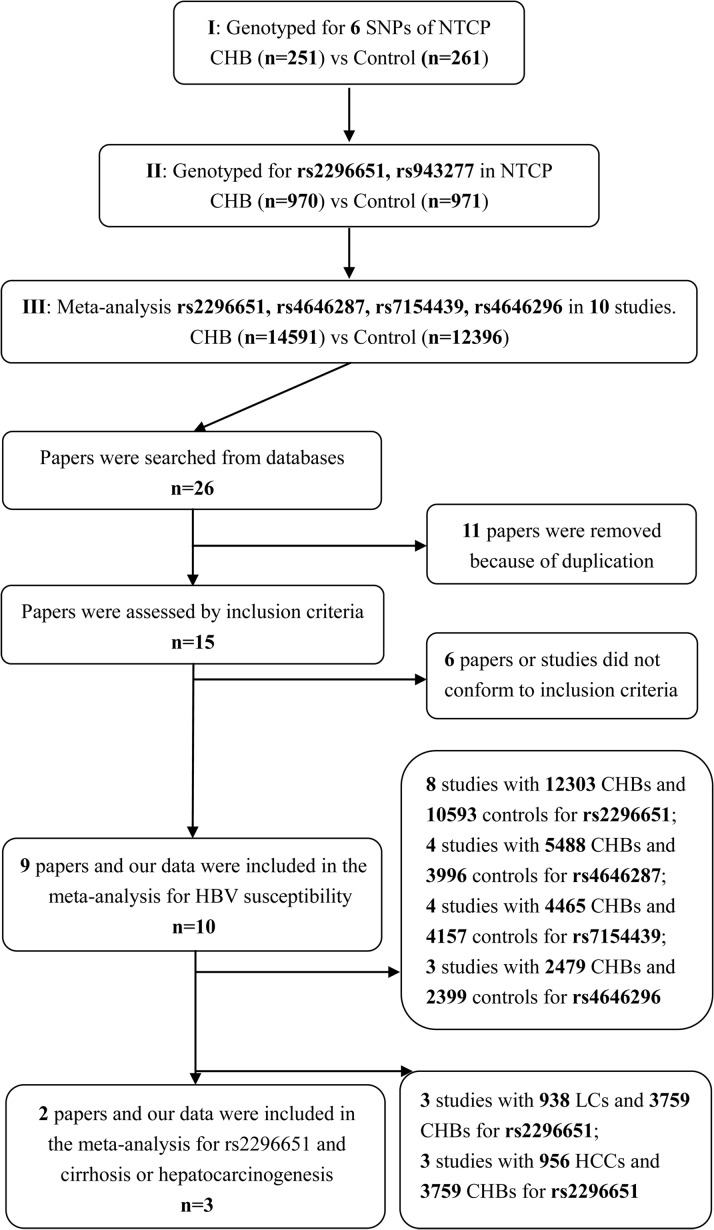
Trial sequential analysis: Gene mutation in NTCP association with HBV infection risk The blue line indicated the Z curve (cumulative effect size), the red curves represented the harm, benefit, and futility boundaries, the red vertical line is the adjusted optimal sample size, the brick-red lines represent the conventional confidence intervals. The black number and marking in the X-axis is the actual number of samples size included in the meta-analysis. The heterogeneity-adjusted sample size based on power of 0.90, (α=5% and β=10%) were 21464, 9066. **(A)** Although the number of the participants had not achieved the required size, the gap is small enough to be ignored (20958 vs 21464) and the cumulative Z-curve had crossed the monitoring boundaries. Sufficient evidence suggested rs2296651-GA genotype negatively association with HBV infection risk. **(B)** The actual samples did not meet the required size for rs4646287 (8178 vs 9066), which the gap was negligible. The cumulative Z-curve had not crossed the monitoring boundaries suggested that the evidence of rs4646287-GA genotype negatively association with HBV infection risk is insufficient.

Meta-analysis was performed to assess the association between rs2296651 and HBV infection, including CHB patients (n=12303) and HCs (n=10593). Although the number of people included was not fully achieved to meet the required sample size to undertake trial sequential analysis (TSA)[20], the gap was small enough to be ignored (actual sample size/required sample size=20958/21464) based on the pre-set 90% power. Furthermore, the cumulative Z-curve had crossed the monitoring boundaries (red lines), confirming our data were reliable (Figure 1).

**Figure 2:**
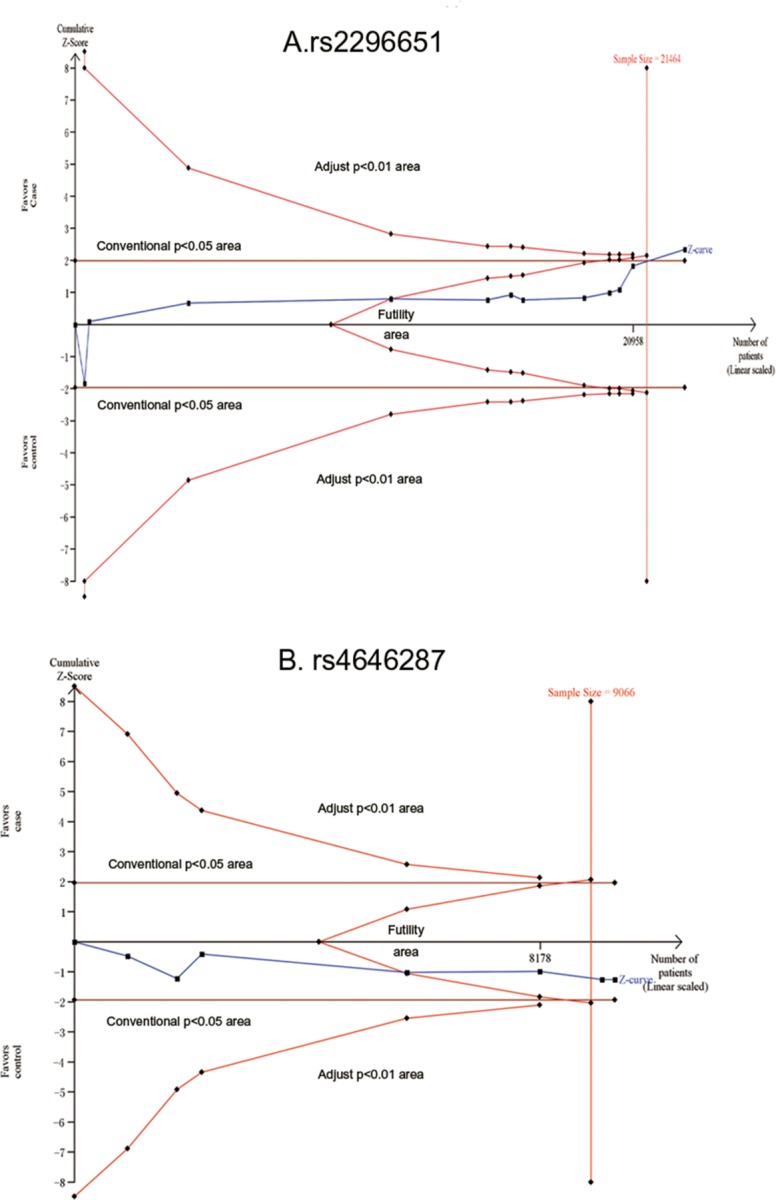
Forest plots show odds ratio (OR) for the associations between NTCP variations (rs2296651) and HBV infection **(A)** allele model (G vs A); **(B)** heterozygote model (GA vs GG); **(C)** recessive model (AA+GA vs GG); **(D)** additive model.

Considering high heterogeneity among the included reports (shown in Table 4 and Figure 2), a random effect model and genetics model analysis were adopted to assess the data. There were lower A allele frequencies in the CHB group (OR=0.607, 95%CI=0.388-0.948, *p*=0.028) compared with HCs group. The GA frequency of rs2296651 in CHB patients was significantly lower than that in HCs. The OR for GA in the codominant model analysis was 0.593 (95%CI=0.376-0.937, *p*=0.025) and in the recessive model was 0.59, (95%CI=0.370-0.941, *p*=0.027). In the overdominant model (GA *vs* GG+AA), the OR was 0.595 and also suggested that the GA frequency was lower in the CHB group. All of these results demonstrated that GA-rs2296651 was inversely correlated with HBV infection.

**Table 4:** Meta-analysis of association between rs2296651, rs4646287, rs7154439, rs4646296 polymorphism and HBV infection

RsID	Model	Comparison	Studies	I^2^ (%)	Model	OR (95% CI)	*p1*	*p2*	*p3*	*p4*
rs2296651	Alleles	G vs A	8	95.3%	Random	**0.607 (0.388, 0.948)**	**0.028**	0.001	0.346	0.468
	Codominant	GA vs GG	8	94.9%	Random	**0.593 (0.376, 0.937)**	**0.025**	0.001	0.394	0.532
	Codominant	GG vs AA	8	87.0%	Random	1.656 (0.232, 11.83)	0.615	0.001	0.692	0.587
	Dominant	GG+GA vs AA	8	86.4%	Random	0.654 (0.096, 4.466)	0.665	0.001	0.695	0.590
	Recessive	GG vs AA+GA	8	95.2%	Random	**0.590 (0.370, 0.941)**	**0.027**	0.001	0.376	0.505
	Overdominant	GG+AA vs GA	8	94.8%	Random	**0.595 (0.378, 0.936)**	**0.025**	0.001	0.399	0.538
	Additive		8	95.3%	Random	**0.607 (0.388, 0.948)**	**0.028**	0.001	0.346	0.468
rs4646287	Alleles	G vs A	4	0.01%	Fixed	0.995 (0.900, 1.102)	0.929	0.432	0.188	0.204
	Codominant	GA vs GG	4	31.9%	Fixed	0.962 (0.860, 1.075)	0.492	0.184	0.064	0.073
	Codominant	GG vs AA	4	0.01%	Fixed	0.769 (0.501, 1.183)	0.232	0.552	0.893	0.595
	Dominant	GG+GA vs AA	4	0.01%	Fixed	1.310 (0.853, 2.012)	0.217	0.534	0.852	0.564
	Recessive	GG vs AA+GA	4	17.5%	Fixed	1.023 (0.917, 1.142)	0.678	0.296	0.101	0.113
	Overdominant	GG+AA vs GA	4	33.3%	Fixed	1.044 (0.933, 1.167)	0.452	0.174	0.062	0.072
	Additive	G vs A	4	0.01%	Fixed	0.995 (0.900, 1.102)	0.929	0.432	0.188	0.204
rs7154439	Alleles	G vs A	4	29.9%	Fixed	1.027 (0.948, 1.112)	0.514	0.189	0.524	0.502
	Codominant	GA vs GG	4	34.2%	Fixed	1.059 (0.962, 1.165)	0.240	0.156	0.988	0.992
	Codominant	GG vs AA	4	26.2%	Fixed	1.053 (0.828, 1.339)	0.672	0.219	0.219	0.227
	Dominant	GG+GA vs AA	4	26.3%	Fixed	0.933 (0.735, 1.185)	0.570	0.219	0.215	0.228
	Recessive	GG vs AA+GA	4	32.2%	Fixed	0.955(0.871, 1.047)	0.330	0.171	0.763	0.747
	Overdominant	GG+AA vs GA	4	34.2%	Fixed	0.942(0.857, 1.036)	0.219	0.155	0.875	0.896
	Additive	G vs A	4	29.9%	Fixed	1.027 (0.948, 1.112)	0.514	0.189	0.524	0.502
rs4646296	Alleles	G vs C	3	0.01%	Fixed	1.018 (0.896, 1.157)	0.781	0.843	0.871	0.873
	Codominant	GC vs GG	3	0.01%	Fixed	1.017 (0.882, 1.171)	0.821	0.823	0.657	0.654
	Codominant	GG vs CC	3	0.01%	Fixed	0.937(0.528, 1.664)	0.825	0.545	0.201	0.233
	Dominant	GG+GC vs CC	3	0.01%	Fixed	1.062 (0.599, 1.883)	0.837	0.541	0.185	0.217
	Recessive	GG vs CC+GC	3	17.5%	Fixed	0.982 (0.854, 1.129)	0.800	0.837	0.870	0.871
	Overdominant	GG+CC vs GC	3	0.01%	Fixed	0.985 (0.855, 1.135)	0.837	0.819	0.604	0.607
	Additive	G vs C	3	0.01%	Fixed	1.018 (0.896, 1.157)	0.781	0.843	0.871	0.873

I^2^, variation in OR attributable to heterogeneity; OR, odds ratio; 95% CI, 95% confidence interval; Random, random effects model; *p1*, p value for OR;*p2*, p value for heterogeneity; *p3, p4*, Egger test and Harbord test for publication bias; Bold values are statistically significant (*p* < 0.05); Na, not available.

As for rs4646287, rs7154439 and rs4646296, a fixed effects model was adopted because low heterogeneity existed among all the included studies (I^2^<50%). Meta-analysis from the combined 3 reports showed no correlation between the rs4646287 polymorphism and HBV infection, including CHB patients (n=5488) and HCs (n=3996) (Table 4 or Supplementary Figure 1). There was no significant difference between CHB patients (n=4465) and HCs (n=4157) for rs7154439 variation (Table 4 or Supplementary Figure 1). Similarly, there was no correlation between rs4646296 mutants and HBV infection in the genetics model analysis (2479 CHBs vs 2399 HCs) (Table 4 or Supplementary Figure 1).

In the trial sequential analysis, sample size of the 3 loci (rs4646287, rs7154439 and rs4646296) did not meet the required level (actual sample size vs required sample size: 8178 vs 9066, 3971 vs 6845 and 2682 vs 7721). The cumulative Z-curve of these variations did not surpass the monitoring boundaries (data not shown).

### Publication bias

**Figure 3:**
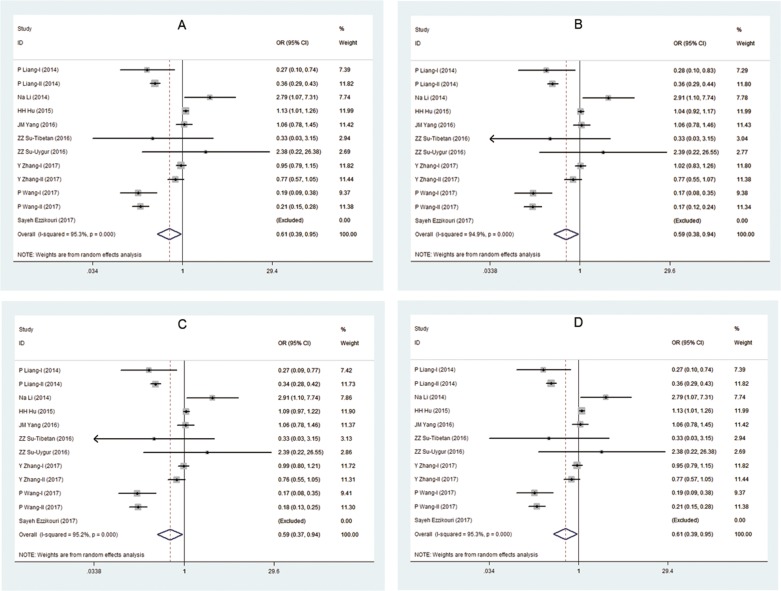
Funnel plot for publish bias in selection of studies on the polymorphism of NTCP and HBV infection (recessive and additive model) **(A, B)** The publish bias between the rs2296651 polymorphism and risk of HBV infection (recessive model) and (additive model). **(C, D)** The publish bias between the rs4646287 polymorphism and risk of HBV infection (recessive model) and (additive model). **(E, F)** The publish bias between the rs7154439 polymorphism and risk of HBV infection (recessive model) and (additive model). **(G, H)** The publish bias between the rs4646296 polymorphism and risk of HBV infection (recessive model) and (additive model).

Although the funnel plot indicated evidence of certain asymmetry because of high heterogeneity, there was no potential publication bias existed among all conclusions of these mutants through calculating Harbord’ test, Egger's test or Begg's test (all *p*>0.05) (Figure 3 and Table 4).

### Grading of GRADE and credibility of meta-analysis results

Based on the Venice Guidelines of Recommendations Assessment, Development and Evaluation of quality, credibility of the association of the variants of NTCP with HBV infection were calculated by GRADEpro software. Due to observational studies, inconsistency risk, small number of studies as well as smaller sample sizes, the quality of evidence for rs2296651, rs4646287, rs7154439 and rs4646296 mutants were graded as moderate quality. Finally, GRADE result demonstrated evidence for the association between rs2296651 variants and HBV infection, and was ranked as moderate and the conclusion was identified as critical (very important) grade. In addition, evidences of rs4646287, rs7154439 and rs4646296 were ranked as moderate and the conclusions were viewed as important grades (as presented in Supplementary Figure 2).

### Inverse association between NTCP rs2296651 mutant and HBV-related LC/HCC

To assess the association between rs2296651 and LC/HCC, patients with LC or HCC were compared with healthy individuals and CHB (patients without liver cirrhosis and HCC), respectively. The frequency of rs2296651-GA was lower in LC or HCC patients [adjusted OR(95%CI) = (0.21-0.55), *p*=0.001; or 0.46(0.25-0.83), *p*=0.008], compared with HCs, using multivariate logistic analysis (Table 5). Logistic analysis also showed that participants with the GA genotype of the rs2296651 mutant had increase risk of being in the LC group (adjust OR (95%CI), which was 2.033(1.083-3.818), *p*=0.027, dominant model), compared with the CHB group (Table 5). However, there were no significantly association of the rs2296651-GA mutant between the HCC group and CHB group [AOR (95%CI) which was 0.889(0.290-2.731), *p*=0.837, dominant model], due to the relatively small sample size (Table 5).

**Table 5:** Association between rs2296651 and LC, HCC in present study

RS ID	Model	Genotype	HC	CHB	LC	HCC	AOR_1_	*P*_1_	AOR_2_	*P*_2_	AOR_3_	*P*_3_	AOR_4_	*P*_4_
rs2296651	Alleles	G	2172(89.3)	1682(98.5)	452(95.4)	229(97.9)	1		1		1		1	
		A	260(10.7)	26(1.5)	22(4.6)	5(2.1)	0.532 (0.328-0.861)	**0.01**	1.95 (1.08-3.521)	**0.026**	0.519 (0.273-0.99)	**0.046**	0.866 (0.292-2.575)	0.796
	Codominant	GG	956(78.6)	828(96.8)	216(90.8)	112(95.7)	1		1		1		1	
		GA	260(21.4)	26(3.0)	20(8.4)	5(4.3)	0.341 (0.211-0.549)	**0.001**	2.934 (1.607-5.358)	**0.003**	0.46 (0.254-0.832)	**0.008**	1.422 (0.535-3.778)	0.484
		AA	0(0)	1(0.1)	1(0.8)	0(0)	NA	NA	NA	NA	NA	NA		
	Dominant	GG+GA	1216(100)	854(99.9)	236(99.2)	117(100)	1		1		1		1	
		AA	0(0)	1(0.1)	1(0.8)	0(0)	NA	NA	2.033 (1.083-3.818)	0.027	NA	NA	0.889 (0.290-2.731)	0.837
	Recessive	AA+GA	260(21.4)	27(3.1)	21(8.8)	5(4.3)	1		1		1		1	
		GG	956(78.6)	828(96.9)	216(91.2)	112(95.7)	0.499 (0.306-0.818)	**0.001**	2.994 (0.186-48.26)	0.44	0.52 (0.273-0.99)	**0.041**	NA	NA
	Additive						0.532 (0.328-0.861)	**0.01**	1.95 (1.08-3.521)	**0.026**	0.519 (0.273-0.99)	**0.046**	0.866 (0.292-2.575)	0.796

LC, liver cirrhosis. HCC, hepatocellular carcinoma. Data were presented as number (percentage) for every group. The differences in genotype frequencies between any two groups were analyzed using logistic regression models (Codominant, Recessive, Dominant, additive). Age and sex were included as covariates. AOR_1_ and *P*_1_, Comparison of patients with LC to HC. AOR_2_ and *P*_2_, Comparison of patients with LC to CHB. AOR_3_ and *P*_3_, Comparison of patients with HCC to HC. AOR_4_ and *P*_4_, Comparison of patients with HCC to CHB. AOR(95% CI), adjusted odds ratio was calculated and reported within the 95% confidence interval. Significant *p*-values (*p* < 0.05) were highlighted in bold.

However, it was observed that the inverse association between mutant rs2296651 and HCC was present, OR is 0.701 (*p*=0.001, recessive model), when analysed by systematic review and meta-analysis (2 studies and our data; characteristics of the included studies are shown in Supplementary Table 5), including HCC patients (n=956) and CHB patients (n=3759), (Table 6). To evaluate the association between rs2296651 and LC, the LC patients (n=938) were compared to the CHB patients (n=3759). Meta-analysis demonstrated no significant association between rs2296651 and the LC patients, which might be due to high heterogeneity (Table 6). The Harbored’ test and Egger’ test of meta-analysis further confirmed no publication bias (Table 6). These results of meta-analysis suggest a significant inversely association between rs2296651-GA genotype and HCC patients, but not cirrhosis.

**Table 6:** Meta-analysis of association between rs2296651 polymorphism and HBV-related LC or HCC

RsID	Patients	Genetic Model	Comparison	Studies	I^2^ (%)	Meta Model	OR (95% CI)	*p1*	*p2*	*p3*	*p4*
rs2296651	**LC**	Alleles	G vs A	3	90.5	Random	1.374 (0.519, 3.643)	0.522	0.001	0.431	0.391
		Codominant	GA vs GG	3	89.7	Random	1.376 (0.514, 3.686)	0.525	0.001	0.415	0.378
		Recessive	GG vs AA+GA	3	90.3	Random	1.380 (0.506, 3.763)	0.529	0.009	0.424	0.385
rs2296651	**HCC**	Alleles	G vs A	3	28.9	Fixed	**0.722 (0.562, 0.837)**	**0.002**	0.245	0.129	0.113
		Codominant	GA vs GG	3	35.0	Fixed	**0.701 (0.564, 0.872)**	**0.001**	0.215	0.129	0.110
		Recessive	GG vs AA+GA	3	35.0	Fixed	**0.701 (0.564, 0.872)**	**0.001**	0.215	0.129	0.110

I^2^, variation in OR attributable to heterogeneity; OR, odds ratio; 95% CI, 95% confidence interval; Random, random effects model; *p1*, p value for OR; *p2*, p value for heterogeneity; *p3, p4 are p_Egger_* and *p_Harbord_* for publication bias; Bold values are statistically significant (*p* < 0.05); Na, not available.

## DISCUSSION

In the present study, NTCP polymorphisms were investigated in CHB and HBV-related HCC patients among the Chinese Han population. The NTCP rs2296651-GA variant was inversely correlated with susceptibility to HBV infection or HBV-related HCC. It was confirmed that individuals who carry the rs2296651-GA genotype have a lower risk of HBV infection than those of the GG genotype, suggesting a possible protective role of this variant (rs2296651-GA) during HBV infection. The association between the rs2296651 mutant and HCC was further investigated, demonstrating that participants with the rs2296651-GA genotype have lower risk of HCC compared with individuals who carry the GG genotype. These associations were then verified using meta-analysis. In addition, the GA genotype of the rs943277 mutant on NTCP has for the first time been revealed to be a potential hazardous mutant, facilitating susceptibility to HBV infection. However, there was no association among other variants (rs17556915, rs4646296, rs9323529, rs943277, rs4646287 and rs7154439) with HBV susceptibility or HCC. Our current study is the first systemic review and meta-analysis that focused on the association between NTCP polymorphisms and HBV susceptibility or HBV-related HCC.

NTCP is a functional receptor for HBV entry *in vitro* [6], which is supported by the findings that HBV entrance is blocked *via* inhibiting NTCP in hepatocyte using cyclosporine A [21] and that tent-making bat HBV (TBHBV) can infect human hepatocytes *via* the human NTCP (hNTCP) receptor [22, 23]. Moreover, the species specificity and hepatotropism of HBV infection were determined by the functional receptor restrictions [24–26]. Our data and other gene association studies [10–13] have further verified the role of NTCP in HBV susceptibility from the perspective of genetics correlation *in vivo*.

HBV susceptibility in certain populations is partially influenced by ethnic difference [27] and gene polymorphism [28]. The rs2296651 (S267F) mutation leads to an amino acid change in human NTCP, resulting in functional alterations of NTCP [28]. The GA mutant on rs2296651 has been identified as an Asia-specific variant among Asia countries [29]. When linked to the high prevalence of HBV infection in Asia, especially in China, it was of great interest to determine the association between the rs2296651 mutant and HBV susceptibility in these regions. However, the association study between rs2296651 polymorphisms of NTCP and HBV susceptibility is controversial. Yang *et al* [13] reported that the variant frequency of the rs2296651 A allele is decreased in HBV carriers, compared to that in HCs. But others have demonstrated that the GA genotype of rs2296651 promotes HBV infection (OR=2.91, *p*=0.03 for GA genotype) [11]. Subsequently, in large sample studies, rs2296651 mutants have been associated with resistance to HBV infection (OR of GA = 0.36, *p*<0.001 and OR of AA = 0.16, *p*<0.001, respectively) [10], (OR of AA = 0.13, *p*<0.001) [12]. In addition, no association of variants have been found at the SLC10A1 locus with susceptibility to persistent HBV infection among Southern Chinese [14].

In the current study, our data show that the proportion of the rs2296651-GA genotype was dramatically lower in CHB patients than in HCs, demonstrating a potential protective effect against HBV infection of the rs2296651-GA variant of NTCP. Our results are in line with the findings of Peng *et al*. [10] and Hu *et al*. [12], showing the low frequencies of rs2296651-GA in CHB patients. Subsequently, a potential protective role of the rs2296651-GA mutation was further confirmed in our current study using meta-analysis, showing the NTCP rs2296651-GA genotype was inversely correlated with HBV infection (OR=0.593, *p*=0.028). Such data demonstrate that individuals with the rs2296651-GA genotype have significantly lower risk of HBV infection than those with the GG genotype.

There have been additional reports on the inverse correlations between the rs2296651 mutant and HBV susceptibility in regions with high minor allele frequency (MAF) [10, 12], while no correlations have been observed in regions with low MAF [13, 14]. These controversial findings might be due to different levels of genetic variations among the Asian population. Regarding the minor allele frequency of rs2296651, MAF is 7.5% in Chinese-Americans [29], 3.1-5% in Koreans, 7.4% in Chinese and 9.2% in Vietnamese [30]. Moreover, among the Chinese population there are different frequencies of the rs2296651 mutant, i.e. 8.3% and 8.1% in Guangdong and Guangxi provinces (Southern China), 7.5% in Chongqing, 5.4% in Hunan and Hubei provinces (Central China) and 2.4% in Henan province (near Northern China)[13]. These reports are consistent with our current data, as well as other meta-analysis [10–13, 16]. However, there is no standard cutoff-value of MAF to accurately estimate HBV susceptibility at present, which needs to be further investigated.

Recently, it is reported that NTCP expression is decreased in HCC tumor tissue [31]. Kang *et al*. further demonstrate that the lower levels of NTCP expression in tumor tissues are associated with a poor post-surgery survival rate in HCC patients [9]. In addition, ectopic expression of NTCP in HCC inhibits tumor cell proliferation and growth [9]. However, the association between NTCP mutants and HCC is still controversial [11, 12]. Our current study has shown that CHB patients with the rs2296651-GA genotype have a significantly decreased HCC risk, basing on meta-analysis of 3 studies (OR is 0.701, *p*=0.001, in dominant model), consistent with results reported by Hu *et al*. [12].

The NTCP rs2296651-GA mutation, a non-synonymous mutation in exon 4 causing an amino acid change, reduces HBV entrance into hepatocytes *in vitro* [28]. It might inhibit HBV viral replication and protect newly differentiated hepatocytes against HBV infection. Disabling this function of NTCP might wipe out the advantages in preventing HBV infection, persistent inflammatory injury and even hepatocarcinogenesis. In addition, NTCP is critical for maintaining homeostasis of bile acid [7, 32]. It had been reported that binding of HBV to NTCP alters bile acid metabolism [33] and that the rs2296651-GA mutant in NTCP is deleterious for the transport of bile acids [29]. The functional limitation of bile acid transportation and the metabolic disorder of bile acid might promote cell death of HBV infected hepatocytes, which would further prevent HBV replication and spread [33]. The metabolic disorder of bile acid and the low HBV replication level, which are likely caused by the rs2296651-GA mutant, might explain the lower occurrence of HCC and the lower HBV cccDNA level in HCC patients with down-regulated expression levels of NTCP [9]. It has been reported that the IL-28B genotype is the strongest pretreatment predictor for sustained virologic response (SVR) in HCV genotype 1 patients based on Peg-IFN-α treatment, because IL-28B polymorphisms influences viral kinetics [34]. Moreover, IL-28B rs12979860 C/T mutant is an important predictive factor in the development of HCC in liver cirrhosis patients [35]. Thus, our findings of NTCP polymorphisms among HBV patients, especially the results of the rs2296651-GA mutant, might also provide an insight for the interaction of HBV and host, and the development of HCC. Recently, in CHB patients with rs2296651-GA mutant (S267F variant), more virological response and sustained response with HBsAg loss at 24 weeks following Peg-IFN-α treatment were found [36]. The S267F variant on the NTCP gene was independently associated with sustained normalization of ALT following treatment with Peg-IFN-α in HBeAg-positive CHB patients [36], which provide additional support for the clinical significance of the rs2296651 beyond HBV entry. Understanding the underlying mechanism between NTCP polymorphisms and HBV infection/HBV related HCC would be helpful in designing a novel therapeutic agent/strategy for CHB.

Rs943277, located in intron 1 of chromosomes 14 in humans, was first genotyped in a Japanese population [37], but has had less research because the potential DNA-binding site for transcription factors was generally believed to be in the promoter region exon of SLC10A1 [37, 38]. Recently, it has been reported that variants in the intron might also influence gene expression via regulating a transcription enhancer or silencer [39]. Moreover, neighboring variants of the promoter region might also lead to missense mutations which could reduce the uptake of bile acid and/or estrone sulfate by NTCP [29]. We undertook this study based on the assumption that rs943277 is close to the promoter region of SLC10A1 [37]. Interestingly, our results of association analysis showed that the frequency of NTCP rs943277-GA genotype was higher in CHB patients than that in HCs (Adjusted OR=2.42, *p*=0.032), as well as the frequency of GA+AA (Adjusted OR=2.32, *p*=0.048). The partial positive correlation demonstrated that rs943277-GA genotype was a potential hazardous variation in HBV susceptibility. It was speculated that the variation at rs943277 might promote HBV infection *via* influencing NTCP expression. Due to limited baseline research on NTCP, especially the rs943277-GA mutant, the function of the rs943277-GA mutant in influencing NTCP expression is still poorly understood [37, 40]. However, our data support an important clue for the functional study of the NTCP gene variation at rs943277. Further work will determine the exact role of the rs943277-GA mutant in the pathogenesis of HBV infection.

As for rs4646287, a functional genetic variant of NTCP is associated with HBV infection [13]. Despite the likelihood that the rs4646287 mutation might reduce the morbidity of HCC [13], there are controversial results for rs4646287 in HBV infection [13, 16, 18]. Our meta-analysis result (OR=0.962, *p*=0.492) supports the view that no firm genetic association has been established for rs4646287 with HBV infection [16]. For the development of HBV infection, it is reported that the rs7154439-AA genotype might be associated with HBV clearance (adjusted OR=0.33, 95%CI=0.15-0.75, *p* =0.008 in a codominant model) [18], but another study reports that the rs7154439 variants are not associated with the risk of HBV infection [19]. In our meta-analysis, rs7154439-AA mutants exhibited no significant difference in distribution between the HC group and CHB group. Similarly, the rs4646296-GC mutant showed no association with HBV infection, consistent with the sporadic data [14, 18] or meta-analysis (OR=1.017. *p*=0.821, codominant model). This disparity might be due to the relatively small sample sizes enrolled in the studies selected in this meta-analysis. As for rs17556915, rs9323529 and rs943276, there were very low minor allele frequency (MAF) value (<1.0%), and no association with HBV infection was found in present study. Considering the relatively small sample size for trial sequential analysis (TSA) or little in the literature, additional studies are needed to determine the correlation between rs4646287, rs7154439, rs464696, rs17556915, rs9323529 or rs943276 and HBV infection/HCC occurrence in the future.

There were several limitations in the present study. Firstly, the selected hot spots might miss other important mutant sites. It would be better to sequence the whole NTCP gene to discover the role of new loci in the pathogenesis of CHB or HCC. Secondly, because of lower homozygosity for rs2296651 than expected in the CHB patients, the distribution of the minor allele was not consistent with the Hardy-Weinberg (H-W) equilibrium (*p*<0.001) in our replication group. A similar Hardy-Weinberg disequilibrium also existed in other large cohorts [10, 12], which could explain the high heterogeneity in the present meta-analysis. Moreover, different genotyping methods and study designs might also contribute to the bias, which could also be the possible reasons for high heterogeneity of included studies in the meta-analysis. Thirdly, the frequencies of rs2296651-GA and rs943277-GA were relatively low in the population both in the patient group and healthy controls. Although meta-analysis has been introduced to confirm the conclusion, further comprehensive investigation using a large-scale multicenter population of varying ethnic origins, with different outcomes of infection, comparable durations of infection and therapeutic schedule, will be performed. Additionally, we were unable to assess the influence of NTCP expression by these mutants in liver tissues, due to limited acquisition of the liver biopsy specimens. However, in our future experiments we will determine the effect of rs943277-GA on the expression of SLC10A1.

In conclusion, the present study provided some polymorphism evidence for the perspective that NTCP gene variations correlate with HBV susceptibility and HBV-related HCC, basing on experimental study and meta-analysis. The most interesting of those variants, rs2296651, might have a potential protective role in HBV susceptibility and hepatocarcinogenesis in regions with high MAF. The role of the partial positive association between rs943277 and HBV susceptibility will also be further investigated. These results might shed some light on the study of HBV infection and the occurrence of HBV-related HCC.

## MATERIALS AND METHODS

### Subjects

The study population included a discovery group and a replication group, with patients assigned according to the recruiting period. A total cohort of 1221 HBV patients in the Southeastern China region were recruited from Jun. 2011 to Dec. 2014 in *the Department of Infectious Diseases, Ruijin Hospital, Shanghai Jiaotong University School of Medicine*. 1232 ethnically and geographically matched healthy controls (HCs) undertaking a routine checkup were recruited from *the Center of Health Examination of Ruijin Hospital* in the same period. For the analysis, 251 HBV outpatients who were investigated before Mar. 2012 were assigned as the discovery group of the study, and the HBV inpatients who were included in the study between June 2012 and Dec 2014 were regarded as the replication group to confirm the association. (Discovery group: Jun. 2011-Mar. 2012, CHB (n=251) and HCs (n=261); Replication group: Aug. 2013-Dec. 2014, CHB (n=970) and HCs (n=971)).

The diagnosis of CHB was established by seropositivity of hepatitis B surface antigen (HBsAg) over a 6-month period according to *the Chinese guideline of prevention and treatment for CHB* (2010 version) [41] and did not have any other type of liver diseases such as chronic hepatitis C, hepatitis D, hepatitis E, drug-induced, autoimmune and alcoholic or nonalcoholic liver diseases. All participants were identified as Han Chinese. The demographic information included gender, age, birth-place, past and current residency. The clinic data were collected from clinical records and/or telephone interviews. The study is approved by the *Ethics Committee of Ruijin Hospital, Shanghai Jiaotong University School of Medicine* in accordance with *the Helsinki Declaration*. The characteristics of CHB, LC, HCC and HC are presented in Table1.

### Single nucleotide polymorphism (SNP) selection

SNPs were selected using HapMap Data Rel 27 Phase II+III, Feb 2009, on NCBI B36 assembly, dbSNP b126 of Han Chinese Beijing (http://hapmap.ncbi.nlm.nih.gov/) and Haploview software 4.2 (Mark Daly's lab at Broad Institute, Cambridge, MA, USA). The criteria used for SNP selection were population-frequency and multiple, high-profile or inconsistent submitters. The core criterion was determined based on alteration of NTCP transcription, translation or function. Six SNPs (rs17556915, rs2296651 [10, 11, 28], rs464629 [18], rs9323529, rs943276 [37] and rs943277 [37]) were finally selected for evaluation. Meta-analysis was performed on rs2296651, rs4646287, rs7154439 and rs4646296. In the discovery group, all the six SNPs were genotyped. In the replication group, only rs2296651 and rs943277 were genotyped, according to the analysis result of discovery group.

### Genomic DNA extraction

Genomic DNA was extracted from 5 ml venous blood, using the DNA Extraction Kit (Tiangen Biotech Co., Ltd., Beijing, China) according to the manufacturer's instructions. After determination of genomic DNA concentration, the samples were stored at −80°C until genetic polymorphism analyses.

### Genotyping

Six SNPs (rs17556915, rs2296651, rs4646296, rs9323529, rs943276 and rs943277) were identified in the region of NTCP gene on chromosome 14 (range from 69310000 to 69335740). SNP ID numbers as well as sequence are available at http://www.ncbi.nlm.nih.gov/snp/ (Supplementary Table 1). The primers used for the corresponding SNPs polymerase chain reaction (PCR) amplification and SNaPshot extension reactions were designed, using Primer 5 software (Supplementary Table 1). SNPs were confirmed by multiplex SNaPshot technology, according to previously described [42], using an ABI fluorescence-based assay allelic discrimination method (Applied Biosystems, Bedford, MA).

PCR was performed as described previously [43, 44]. Briefly, in a total volume of 20 μlcontaining 1×ExTaq 0.2 μl, 25 Mm MgCl_2_ 2 μl, 25 mMdNTP Mix 2 μl, (TaKaRa Bio, Dalian, China), 2 μl genomic DNA, and 4 μl of each primer. The PCR product was purified by 1 U SAP (shrimp alkaline phosphatase) and 1 U Exonuclease I. The product was processed according to the ABI SNaPshot protocol. Extension was performed in a total volume of 10μl containing 5μl SNaPshot Multiplex Kit (ABI), 2μl PCR product, 1 μl mixed extension primer and 2 μl H_2_O. The samples were put through 28 cycles of denaturation at 96°C, annealing at 50°C, elongation a 60°C, and a final extension at 72°C. The extension product was purified by 1 U SAP. The SNP was confirmed using an ABI3130 genetic analyzer. Genotypes were determined automatically using Genemapper 4.0 software (Applied Biosystems).

### Meta-analysis on association of NTCP with HBV infection

**Figure 4:**
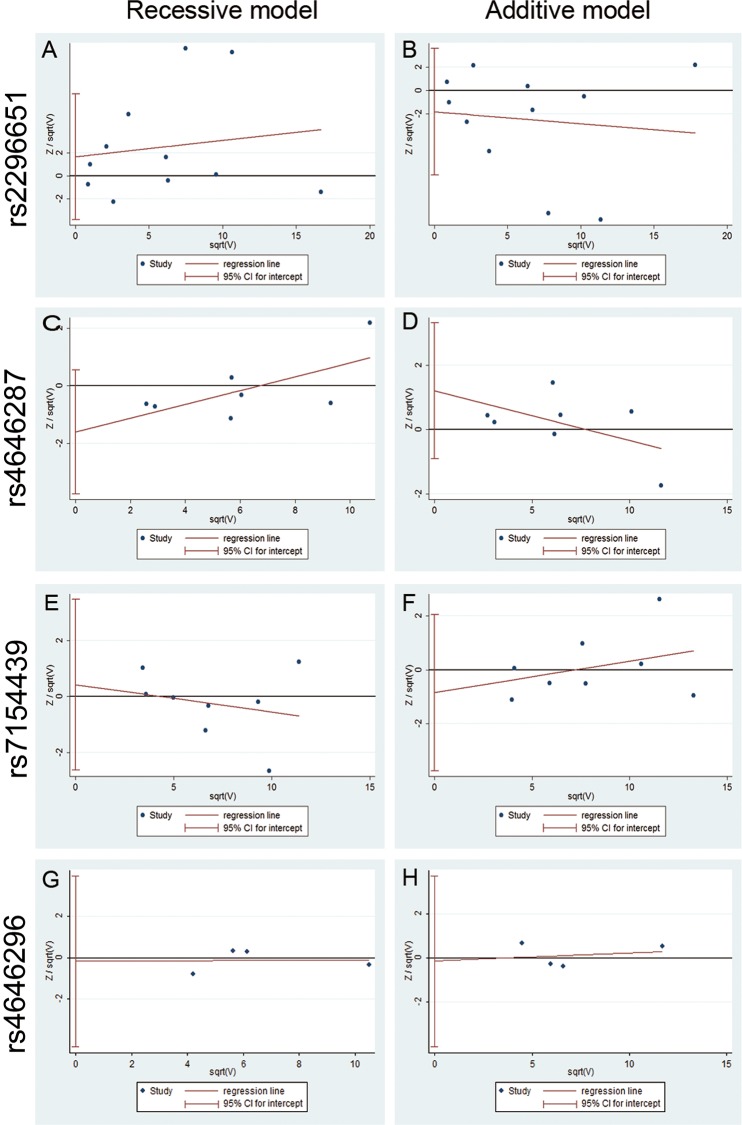
Grouping strategy for the population in the experimental study and selection for eligible studies included in this meta-analysis

Pubmed, Embase, Web of Science and WanFang databases were used to search for SNPs related studies. Search words included sodium taurocholate cotransporting polypeptide or “NTCP” or “SLC10A1” or “S267F” or “rs2296651” or “rs4646287” or “rs7154439” or “rs17556915” or “rs4646296” or “rs9323529” or “rs943277” or “rs943276” and “hepatitis B” or “hepatitis B virus” or “HBV” or “HBV infection” or “CHB” or “liver cirrhosis” or “LC” or “Hepatocellular Carcinoma” or “HCC”. Data were collected by three investigators (Peng Wang, Ruidong Mo and Xiaogang Xiang) (Figure 4).

The inclusion criteria were listed as follows: 1) Article focuses on an association between the polymorphism of NTCP or SLC10A1 and hepatitis B virus or HBV infection or CHB or LC or HCC; 2) Study design is a case-control study; 3) CHB diagnosis meets *The Chinese Guideline of Prevention and Treatment for Chronic Hepatitis B* (2010 version) [41] or *The American Association for the Study of Liver Disease* [45]; 4) Odds ratios with the 95% confidence interval (95%CI) and allele frequencies are available. Using the Newcastle-Ottawa Scale (NOS), three criteria were applied to evaluate the quality of each study for meta-analysis, as described [46]: 4 stars for selection, 2 stars for comparability and 3 stars for exposure/outcome. Nine stars were regarded as the highest quality and more than six stars were deemed as a high quality study.

### Statistical analysis

The significance was determined using student's t test or Z test in demographic and clinical data for two groups or continuous variables. The χ^2^ tests or the Fisher exact tests (two-sided) were used to compare the categorical variables. The differences between groups were examined using the respective genetics models of codominant, dominant, recessive and additive, as appropriate. Statistical significance was performed using the SPSS software version 17.0 (SPSS Inc., Chicago, IL, USA) and GraphPad Prism 5 (GraphPad Software Inc. CA, USA). Hardy-Weinberg disequilibrium, the odds ratios with a 95% confidence interval (95%CI), logistic regression adjusted for age and gender were calculated by PLINK (v.1.07, http://pngu.mgh.harvard.edu/purcell/plink/, 5 February 2015, date last accessed) [47].

Systematic review and meta-analysis were adopted to comprehensively evaluate the correlation of rs2296651 or rs4646287, rs7154439, rs4646296 and HBV susceptibility, and the association of rs2296651 and LC or HCC. The heterogeneity for the included articles was evaluated using Cochran's Q test and I^2^ statistics, which varied from 0 to 100%. The heterogeneity was described as low (0-40%), moderate (30-60%), substantial (50-90%), and considerable (75-100%). Then, a fixed effect model or random effect model was applied, basing on the heterogeneity and Cochran's Q test. Additionally, Harbord's test or Egger's test or Begg's test was used to detect potential publication bias [48], using STATA (version 13.0, STATA Corp LP, College Station, Texas, USA). A two-sided *p* value, *p*< 0.05 was considered as statistically significant.

Larger sample size by integrating data of multiple different experiments could increase the power of meta-analysis, and may also increase random errors, which were the risks of reliability and significance of the meta-analysis. To evaluate whether there is enough power to reach firm conclusions and avoid misleading results from random errors, primary outcomes were analyzed by trial sequential analysis (TSA) [20] (TSA, version 0.9; Copenhagen Trial Unit, Copenhagen, Denmark, 2011). Prior to analysis, 5% risk of a type I error, 90% of power, the O'Brien-Fleming function and relative risk reduction based on low bias were preset, according to the required information size in the TSA analysis. Sequential monitoring boundaries were utilized, including the required information size, the conventional significance boundaries (Z=±1.96), and the trial sequential monitoring boundaries (TSBM). A series of cumulative Z-curve are intercalated to evaluate the information and the required information size was further adjusted according to heterogeneity index score (HIS). The firm evidences of benefits and harmfulness were assumed if the Z-curve crossed the conventional boundaries and TSBM and the required HIS [49].

In addition, we also assessed the quality and robustness of the outcomes, using the methodology of Grading of Recommendations Assessment, Development, and Evaluation (GRADE) [50] (GRADEpro v3.6, the Cochrane Collaboration). According to a series of grading standards, it was determined whether the effect or association was specific. Large effect, plausible confounding would change the effect and dose-response gradient would be upgraded to some extent. Possible limitations in design, risk of bias, high heterogeneity or inconsistency, indirectness of evidence, imprecision of results, or high probability of publication bias would be downgraded. Randomized controlled trials are categorized as high quality, but non-RCT could be reduced. Finally, the importance of evidence was graded as high, moderate, low or very low grade and the quality of conclusion was classed as critical, important or low important for decision making. The importance and qualities of the conclusion in the present study are presented in Supplementary Figure 2.

## SUPPLEMENTARY MATERIALS FIGURES AND TABLES




