# Supplementary material for: Genetic variations of NTCP are associated with susceptibility to HBV infection and related hepatocellular carcinoma
Source: Oncotarget. 2017 Oct 31;8(62):105407–24. doi: 10.18632/oncotarget.22211 (PMC5739647; doi:10.18632/oncotarget.22211)
Supplement: Supplementary file 1 [file oncotarget-08-105407-s001.pdf]

Genetic variations of NTCP are associated with susceptibility to HBV infection and related hepatocellular carcinoma

SUPPLEMENTARY MATERIALS

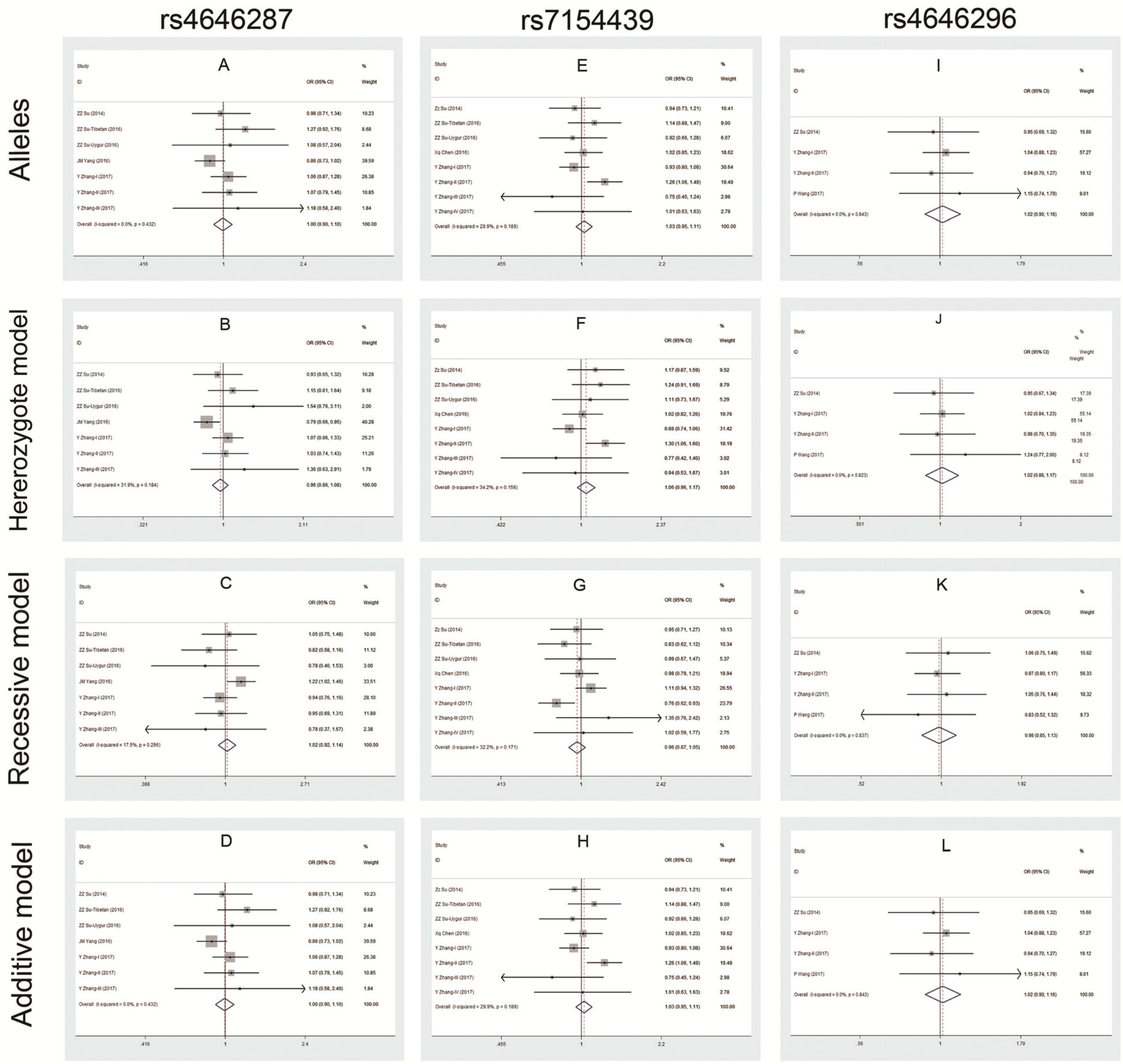

Supplementary Figure 1: Forest plots show odds ratio (OR) for the associations between NTCP variations (rs4646287, rs7154439 and rs4646296) and HBV infection. **(A, E, I)** allele model (G vs A); **(B, F, J)** heterozygote model (GA vs GG); **(C, G, K)** recessive model (AA+GA vs GG); **(D, H, L)** additive model.

Author(s): Peng Wang, Ruidong Mo.
Date: 2017-05-12
Question: Should gene mutations in NTCP be used for HBV infection?
Settings: inpatient, outpatient, in China
Bibliography:

Quality assessment							No of patients		Effect		Quality	Importance
No of studies	Design	Risk of bias	Inconsistency	Indirectness	Imprecision	Other considerations	Gene mutations in NTCP	Control	Relative (95% CI)	Absolute		
rs2296651 association (timing of exposure 3 years; assessed with: genotype)												
8	observational studies[1]	no serious risk of bias	very serious[1]	no serious indirectness	no serious imprecision	very strong association reduced effect for RR >> 1 or RR << 1[1]	12303 cases 10593 controls		OR 0.590 (0.370 to 0.941)	-	⊕⊕⊕O MODERATE	CRITICAL
								15.26%		57 fewer per 1000 (from 8 fewer to 90 fewer)		
rs4646287 association (timing of exposure 3 years; assessed with: genotype)												
4	observational studies[1]	no serious risk of bias	no serious inconsistency	no serious indirectness	no serious imprecision	increased effect for RR ~1[1]	5488 cases 3996 controls		OR 1.023 (0.917 to 1.142)	-	⊕⊕⊕O MODERATE	IMPORTANT
								0.7%		0 more per 1000 (from 1 fewer to 1 more)		
rs7154439 association (timing of exposure 3 years; assessed with: genotype)												
4	observational studies[1]	no serious risk of bias	no serious inconsistency	no serious indirectness	no serious imprecision	increased effect for RR ~1[1]	4465 cases 4157 controls		OR 0.955 (0.871 to 1.047)	-	⊕⊕⊕O MODERATE	IMPORTANT
								25.82%		9 fewer per 1000 (from 26 fewer to 9 more)		
rs4646296 association (Copy) (timing of exposure 3 years; assessed with: genotype)												
3	observational studies[1]	no serious risk of bias	no serious inconsistency	no serious indirectness	no serious imprecision	increased effect for RR ~1[1]	2479 cases 2399 controls		OR 0.982 (0.854 to 1.129)	-	⊕⊕⊕O MODERATE	IMPORTANT
								25.82%		3 fewer per 1000 (from 29 fewer to 24 more)		

[1] case-control

Supplementary Figure 2: Grading of Recommendations Assessment Development and Evaluation (GRADE) the meta-analysis results of gene mutations in NTCP association with HBV infection.

Supplementary Table 1: PCR primers sequence for NTCP genotyping

Rs ID	SNP Property	Base Pair Change	PCR Primers
rs17556915	intron	G>A	F: GGTCAGTTCAGCAGTAGCAG
			R: GATCCCACCATTTCATTGAC
			S: CACCATTTCATTGACTCAACA
rs2296651	missense	G>A	F: TGACTTCAGGTGGAAAGGC
			R: GGAGACTGGATGCCAAAATG
			S: CCAAAATGTCCAACTCTGTT
rs4646296	intron	G>C	F: GCCTACACCACGGACAAGAA
			R: AGGCAGGAAAGATCTTCGAG
			S: TCCTCCAACCCCCAATTGAT
rs9323529	intron	T>G	F: GTCTTGAGAGCTAAAGATCC
			R: TCTAGACCAACCAAATTCCC
			S: AGTATTGTCCTTTGAGGTCAC
rs943276	intron	G>A	F: TACAGCTGGCCCTTTGACAT
			R: GTGAAATCAGCAACTCATTC
			S: CATTCCATTTTTTTAAATGTG
rs943277	intron	G>A	F: CCTGGCTGTTTCTTATAATCG
			R: TTCATGTGCTGCCAGACTTC
			S: AATCCCATTCCAATTTGGGA

Supplementary Table 2: The call rate, Hardy-Weinberg Equilibrium test and frequencies for the 6 SNPs in the study

Rs ID	Chr	Position	MA	Group	Call rate (%)			HWE*		Freq(A1)	
					Controls	Patients	overall	Controls	Patients	Controls	Patients
rs17556915	14	70248357	A	Discovery group	100	99.6	99.8	1	1	0.003	0.01
rs4646296	14	70243504	C	Discovery group	99.6	99.6	99.6	0.676	0.704	0.08	0.092
rs9323529	14	70243812	G	Discovery group	99.6	100	99.8	1	1	0.008	0.004
rs943276	14	70260929	A	Discovery group	99.2	99.6	99.4	1	1	0.008	0.002
rs2296651	14	70245192	A	Discovery group	96.5	100	98.2	1	0.143	0.097	0.02
rs943277	14	70261324	A	Discovery group	99.2	99.6	99.4	1	0.195	0.008	0.017
rs2296651	14	70245192	A	Replication group	100	99.7	99.9	0.09	<0.001	0.109	0.024
rs943277	14	70261324	A	Replication group	99.6	99.9	99.8	1	1	0.002	0.003

Chr: Chromosome; MA: Minor Allele; * Hardy-Weinberg Equilibrium test; Freq: Frequency of A1.

Supplementary Table 3: Quality assessment for included studies using the Newcastle-Ottawa Scale

Study	Year	Selection				Comparability		Exposure			Score
		1	2	3	4	1	2	1	2	3	
Zhenzhen Su	2014	☆	☆	☆	☆	☆	/	☆	☆	/	7
Liang Peng	2014	☆	☆	☆	☆	☆	/	☆	☆	/	7
Na Li	2014	☆	☆	☆	☆	☆	/	☆	☆	/	7
Qiang Li	2015	☆	/	☆	/	☆	/	☆	☆	/	5
Hui-Han Hu	2015	☆	☆	☆	☆	☆	/	☆	☆	/	7
Jingmin Yang	2016	☆	☆	☆	☆	☆	/	☆	☆	/	7
Zhenzhen Su	2016	☆	☆	☆	☆	☆	/	☆	☆	/	7
Xueqin Chen	2016	☆	☆	☆	☆	☆	☆	☆	☆	/	8
Ying Zhang	2017	☆	☆	☆	☆	☆	/	☆	☆	/	7
Sayeh Ezzikouri	2017	☆	☆	☆	☆	☆	/	☆	☆	/	7
Peng Wang	2017	☆	☆	☆	☆	☆	/	☆	☆	/	7

Criteria for Selection: 1) Adequate definition of the cases; 2) Consecutive or obviously representativeseries of cases; 3) Representative controls; 4) No history of investigated diseases for control. Criteria for Comparability: 1) Study controls for age and sex; 2) Study controls for additional important factors, for example, smoking, alcohol, etc. Criteria for Exposure: 1) Exposure ascertainment by blinded review or record; 2) Same method ascertaining exposures in both case and control; 3) Same non-response rate in both groups.

Supplementary Table 4: Characteristics of included studies in meta-analysis of association between rs2296651, rs4646287, rs7154439, rs4646296 polymorphism and HBV infection

See Supplementary File 1

Supplementary Table 5: Characteristics of included studies in meta-analysis of association between rs2296651 mutants and HBV-related LC or HCC

Rs ID	Author	Year	Eehnicity	Method	P_1	P_2	P_3	Genotype	HC	CHB	LC	HCC
rs2296651	N Li#	2014	Northwestern China Han	PCR-RFLP	0.81	0.324	0.324	GG	108	107	56	52
								GA	5	13	9	7
								AA	0	0	0	0
	H Hu#	2015	Taiwan Area	TaqMan	NO	NO	NO	GG	3145	2274	544	679
								GA	651	510	92	101
								AA	5	5	0	0
	P Wang#	2017	Southeastern China Han	SNaPShot	0.032	0.02	0.01	GG	956	828	216	112
								GA	260	26	20	5
								AA	0	1	1	0

#: case-control study design and the blood specimens; CHB: chronic hepatitis B; LC: liver cirrhosis; HCC: hepatocellular carcinoma; $P_{HWE:}$ p value for Hardy-Weinberg equilibrium. P_1, P_2 and P_3 were calculated for CHB, LC and HCC group.
