# Supplementary material for: Genetic variations of NTCP are associated with susceptibility to HBV infection and related hepatocellular carcinoma
Source: Oncotarget. 2017 Oct 31;8(62):105407–24. doi: 10.18632/oncotarget.22211 (PMC5739647; doi:10.18632/oncotarget.22211)
Supplement: Supplementary file 2 [file oncotarget-08-105407-s002.docx]

| **Supplementary Table 4. Characteristics of included studies in meta-analysis of association between rs2296651, rs4646287, rs7154439, rs4646296 polymorphism and HBV infection.** | | | | | | | | | | | | | | | |  |
| --- | --- | --- | --- | --- | --- | --- | --- | --- | --- | --- | --- | --- | --- | --- | --- | --- |
| **RS ID** | **Num** | **Author** | **Year** | **Country or Area** | **Eehnicity** | **Type** | **Sample** | **Method** | **Case** | | | **Control** | | | ***P*_HWE_** | |
|  |  |  |  |  |  |  |  |  | **GG** | **GA** | **AA** | **GG** | **GA** | **AA** |  |  |
| rs2296651 | 1 | Liang Peng-I | 2014 | Southern China | Han | Case-control | blood | Sanger | 94 | 5 | 0 | 75 | 14 | 1 | <0.0001 | |
|  | 1 | Liang Peng-II | 2014 | Southern China | Han | Case-control | blood | Sanger | 1745 | 149 | 5 | 1454 | 348 | 26 | <0.0001 | |
|  | 2 | Na Li | 2014 | Northwestern China | Han | Case-control | blood | PCR-RFLP | 215 | 29 | 0 | 108 | 5 | 0 | 0.324 | |
|  | 3 | Hui-Han Hu | 2015 | Taiwan Area | Taiwan | Case-control | blood | TaqMan | 3099 | 665 | 37 | 3145 | 651 | 5 | NO | |
|  | 4 | Jingmin Yang | 2016 | Eastern China | Han | Case-control | blood | TaqMan | 2419 | 145 | 0 | 1010 | 57 | 0 | NO | |
|  | 5 | Zhenzhen Su | 2016 | Western China | Tibetans | Case-control | blood | PCR | 438 | 1 | 0 | 429 | 3 | 0 | 0.942 | |
|  | 5 | Zhenzhen Su | 2016 | Western China | Uygurs | Case-control | blood | PCR | 195 | 2 | 0 | 233 | 1 | 0 | 0.974 | |
|  | 6 | Ying Zhang-I | 2017 | Southern China Han | Han | Case-control | blood | PCR | 1018 | 251 | 1 | 832 | 201 | 8 | 0.32 | |
|  | 6 | Ying Zhang-II | 2017 | Southern China Han | Han | Case-control | blood | PCR | 223 | 63 | 0 | 477 | 175 | 3 | 0.002 | |
|  | 7 | Sayeh Ezzikouri | 2017 | Moroccan | Moroccan | Case-control | blood | PCR | 286 | 0 | 0 | 109 | 0 | 0 | NO | |
|  | 8 | Peng Wang-I | 2017 | Southeastern China | Han | Case-control | blood | SNaPShot | 241 | 10 | 0 | 203 | 49 | 0 | 0.347 | |
|  | 8 | Peng Wang-II | 2017 | Southeastern China | Han | Case-control | blood | SNaPShot | 921 | 44 | 2 | 758 | 213 | 0 | <0.0001 | |
| rs4646287 | 1 | Zhenzhen Su | 2014 | Southwestern China | Han | Case-control | blood | PCR | 478 | 96 | 7 | 287 | 62 | 3 | 0.862 | |
|  | 2 | Zhenzhen Su | 2016 | Western China | Tibetans | Case-control | blood | PCR | 354 | 79 | 6 | 361 | 70 | 1 | 0.208 | |
|  | 2 | Zhenzhen Su | 2016 | Western China | Uygurs | Case-control | blood | PCR | 178 | 19 | 0 | 216 | 15 | 3 | <0.001 | |
|  | 3 | Jingmin Yang | 2016 | Eastern China | Han | Case-control | blood | TaqMan | 2089 | 423 | 32 | 843 | 215 | 9 | NO | |
|  | 4 | Ying Zhang-I | 2017 | Southern China Han | Han | Case-control | blood | PCR | 1036 | 223 | 16 | 869 | 175 | 13 | 0.21 | |
|  | 4 | Ying Zhang-II | 2017 | Southern China Han | Han | Case-control | blood | PCR | 294 | 66 | 3 | 597 | 130 | 3 | 0.19 | |
|  | 4 | Ying Zhang-III | 2017 | Southern China Han | Han | Case-control | blood | PCR | 74 | 15 | 0 | 107 | 16 | 1 | 0.49 | |
| rs7154439 | 1 | Zhenzhen Su | 2014 | Southwestern China | Han | Case-control | blood | PCR | 405 | 166 | 10 | 249 | 87 | 16 | 0.024 | |
|  | 2 | Zhenzhen Su | 2016 | Eastern China | Tibetans | Case-control | blood | PCR | 308 | 117 | 14 | 319 | 98 | 15 | 0.035 | |
|  | 2 | Zhenzhen Su | 2016 | Eastern China | Uygurs | Case-control | blood | PCR | 125 | 66 | 6 | 149 | 71 | 14 | 0.167 | |
|  | 3 | Xueqin Chen | 2016 | Central China | Han | Case-control | blood | PCR | 503 | 191 | 19 | 726 | 271 | 26 | NO | |
|  | 4 | Ying Zhang-I | 2017 | Southern China Han | Han | Case-control | blood | PCR | 864 | 372 | 48 | 687 | 335 | 37 | 0.69 | |
|  | 4 | Ying Zhang-II | 2017 | Southern China Han | Han | Case-control | blood | PCR | 679 | 349 | 43 | 507 | 200 | 23 | 0.51 | |
|  | 4 | Ying Zhang-III | 2017 | Southern China Han | Han | Case-control | blood | PCR | 62 | 25 | 2 | 78 | 41 | 5 | 1 | |
|  | 4 | Ying Zhang-IV | 2017 | Southern China Han | Han | Case-control | blood | PCR | 65 | 23 | 3 | 144 | 54 | 5 | 1 | |
| rs4646296 | 1 | Zhenzhen Su | 2014 | Southwestern China | Han | Case-control | blood | PCR | 480 | 101 | 0 | 288 | 64 | 0 | 0.061 | |
|  | 2 | Ying Zhang-I | 2017 | Southern China Han | Han | Case-control | blood | PCR | 951 | 310 | 23 | 791 | 253 | 15 | 0.35 | |
|  | 2 | Ying Zhang-II | 2017 | Southern China Han | Han | Case-control | blood | PCR | 296 | 66 | 2 | 587 | 134 | 7 | 1 | |
|  | 3 | Peng Wang-I | 2017 | Southeastern China | Han | Case-control | blood | SNaPShot | 205 | 44 | 1 | 220 | 38 | 2 | 0.676 | |

*P*_HWE:_ value for Hardy-Weinberg equilibrium
